# Association of Diabetes with Meningitis Infection Risks: A Systematic Review and Meta-Analysis

**DOI:** 10.1155/2022/3996711

**Published:** 2022-12-09

**Authors:** Moses Asori, Ali Musah, Razak M. Gyasi

**Affiliations:** ^1^Department of Geography and Earth Science, University of North Carolina, Charlotte, USA; ^2^School of Public Health, Department of Epidemiology and Biostatistics, Kwame Nkrumah University of Science and Technology, Kumasi, Ghana; ^3^Ghana Health Service, Department of Public Health, Regional Health Directorate, Upper West, Ghana; ^4^African Population and Health Research Center, Nairobi, Kenya; ^5^National Centre for Naturopathic Medicine, Faculty of Health, Southern Cross University, Lismore NSW, Australia

## Abstract

**Background:**

The Global Burden of Disease Study in 2016 estimated that the global incident cases of meningitis have increased by 320,000 between 1990 and 2016. Current evidence suggests that diabetes may be a prime risk factor for meningitis among individuals, including older adults. However, findings of prior studies on this topic remain inconsistent, making a general conclusion relatively difficult. This study aimed to quantitatively synthesize the literature on the risk of meningitis associated with diabetes and compare the risk across different global regions.

**Method:**

Literature search and study design protocol followed the Preferred Reporting Items for Systematic Reviews and Meta-Analyses (PRISMA) guidelines. The search was conducted in PubMed, Web of Science, African Journal Online, and Google Scholar using relevant MESH terms. A random effect model was used to pull effect sizes.

**Results:**

Initial search yielded 772 papers but 756 studies were excluded due to duplicity and not meeting inclusion criteria. In all, 16 papers involving 16847 cases were used. The pulled effect size (ES) of the association between diabetes and meningitis was 2.240 (OR = 2.240, 95% CI = 1.716–2.924). Regional-base analysis showed that diabetes increased the risk of developing meningitis in Europe (OR = 1.737, 95% CI = 1.299–2.323), Asia (OR = 2.192, 95% CI = 1.233–3.898), and North America (OR = 2.819, 95% CI = 1.159–6.855). These associations remained significant in the study design and etiological classe-based subgroup analyses. However, we surprisingly found no studies in Africa or South America.

**Conclusion:**

Diabetes is a risk factor for developing meningitis. Given that no research on this topic came from Africa and South America, our findings should be contextually interpreted. We, however, encourage studies on diabetes-meningitis linkages from all parts of the world, particularly in Africa and South America, to confirm the findings of the present study.

## 1. Introduction

According to the Global Burden of Disease Study, the global incident cases of meningitis have increased from 2.50 million (95% uncertainty interval (UI): 2.19–2.91) in 1990 to 2.82 million (2.46–3.31) in 2016 [1]. In this review, we define meningitis in its general term, which may include all meningitis caused by virus, bacteria, and/or fungi. Meningitis is a deadly inflammatory infection that affects the meninges (membranes) of the spinal cord and the brain. Meningitis can be caused by bacteria, viruses, fungi, and other non-infectiousagents such as medications, malignancy, and autoimmune diseases [1–5] making its etiologic nature relatively complex. Bacterial meningitis has been reported as the most prevalent meningitis disease globally, with about 1.2 million cases reported annually across the world [6]. Meningitis has high mortality rates; even among survivors, one in five may develop permanent sequelae such as cranial nerve palsies, hydrocephalus, seizures, hemiparesis, and visual and hearing impairment [1, 7]. Even though the mortality rate has declined by 21% between 1990 and 2016, especially with the postintroduction of *Hib* and *S. pneumoniae* vaccines [1]. However, meningitis remains a significant health challenge in low- and middle-income countries, demanding urgent and comprehensive public health attention. Between 2005 and 2007, the US recorded 4100 cases of bacterial meningitis [8], and the incident rate in Europe could range between 2.6–6.0 per 100,000 people [9]. This incident rate could be 10 times higher in resource-poor settings such as sub-Saharan Africa (SSA) and poorer countries in Asia [10]. For example, a study conducted in Tanzania reported a bacterial meningitis prevalence of 18.6% [11]. Typically, cryptococcal meningitis which is fungal-based has been discovered as the most prevalent among people living with HIV (PLHIV). Therefore, HIV-endemic nations may face significant threat from cryptococcal meningitis.

Approximately 290,000 people lost their lives in 2015 due to meningitis infection [12]. However, the mortality rate may vary with geography, age, sex, smoking/alcoholism prevalence, and underlying acute/chronic medical conditions such as diabetes mellitus and cancer [1, 4, 13–16]. If meningitis remains untreated, its fatality rate can exceed 70% (CDC, (accessed), 2022), with the general mortality rate being 30 and 7% for *S. pneumoniae* and *N. meningitidis*, respectively, in more developed countries [17, 18]. The development and prevalence of these bacterial, viral, fungal, and noninfectious meningitis (including, their negative outcomes in patients) can be moderated by medical and behavioral conditions such as smoking, alcoholism, and built-up conditions. For an example, Iles et al. [19] found a significant association between high levels of passive exposure to tobacco smoke and bacterial meningitis in Australian children. In Poland, smoking was significantly associated with negative outcomes in people suffering from bacterial meningitis (BM) [16]. For example, alcoholism was associated with a lower Glasgow Coma Scale (GCS) (*p* = 0.036) and the presence of seizures (*p* = 0.041) [16].

Type 1 and 2 diabetes have been associated with multiple infectious diseases such as tuberculosis and pneumonia [20]. For example, 80% of individuals who are infected with *Mycobacterium tuberculosis* will not develop the disease until they are influenced by a noncommunicable disease such as diabetes [21]. Particularly, diabetes has been associated with a risk ratio of 1.21 for the development of infections and 2.17 for hospitalization [20]. Some researchers suggest that diabetes may result in a serious immunocompromised state in patients [22–24]. As a result, there is a higher propensity for reduced cell-mediated immune activities and dysfunctioning of the polymorphonuclear leukocytes, monocytes, and lymphocytes; all these have been associated with the magnitude and persistency of hyperglycaemia in patients [25–27]. Even though studies reporting on this association are few, with most recent papers being short case reports, the strength of the relationship has not been consistent across studies. For example, a study in China found no association between diabetes and the prevalence of *C. neoformans* meningitis [28]. A similar study conducted in Spain found no association between diabetes and bacterial meningitis [29]. On the contrary, Chen et al. [30] found diabetes as a risk factor in the development of meningitis. Due to further etiological complications of meningitis, even the association between diabetes and specific subtypes of meningitis may not be consistent for the same population group. For instance, in a Spanish study, it was noted that diabetes mellitus was the strongest correlate of *S. pneumoniae* risk, followed by *L. monocytogenes* [31]. Conversely, age, pregnancy, an underlying cancer condition, and immunosuppression were significant predictors of *L. monocytogenes* instead of *S. pneumoniae* [31]. Since the advent of vaccines for managing meningitis, the etiological spectrums of the various pathogenic agents have changed significantly, thus making their association with underlying comorbidities or risk factors increasingly complex [31]. Considering such inconsistent and disparate findings, there is a need to provide a quantitative summary of the evidence on the state of association between diabetes and meningitis to inform public health decision and policy development. However, to our knowledge, a meta-analysis providing such integrated evidence is limited. This study, therefore, uses a meta-analytic and systematic review protocol to provide synthesized evidence on the impact of diabetes in the development of meningitis.

## 2. Methods

This quantitative review was conducted using the PRISMA (preferred reporting items for systematic reviews and meta-analyses) guidelines [32].

### 2.1. Databases and Search Criteria

A comprehensive literature search was conducted by MA and AM in recognized databases of PubMed and Web of Science, African journal online (Ajol), and Google Scholar. MESH terms (see Table 1) together with appropriate Boolean operators were used to extract papers from recognized databases.

We also further screened downloaded papers for additional papers. All extracted papers were managed using Mendeley reference manager.

### 2.2. Inclusion and Exclusion Criteria

Studies were included only if they reported the association between meningitis and diabetes (see Table 1 for MESH terms). All studies that reported exclusively on meningitis or diabetes without any relationship between them were excluded since it would be difficult to conduct a comparative risk analysis. All in vitro and in vivo studies were also excluded. There were no year restrictions on papers eligibility. We included only peer-reviewed journal articles reporting on primary findings, and there were no restrictions on the type of study design used unless the study was an animal-model study. In this regard, all editorials, letter to editors, correspondences, review papers, working papers, policy briefs, case reports, and theses/dissertations were excluded. We did not impose any geographic restriction on studies except that the study/studies was/were in languages other than English (due to financial constraints for translation services). Moreover, we considered all primary studies that reported odd ratios, adjusted odd ratios, relative risk, risk ratios, or statistical information which made it possible to compute the odd ratios for the meta-analysis. Since the subject we are investigating (impact of diabetes on meningitis) is not commonly researched, we did not impose restrictions on sample size. In other words, all studies independent of sample size were included. Initially, paper titles were screened independently by MA and AM to exclude clearly ineligible articles before downloading. After downloads, abstracts of various papers were further screened to check for further eligibility before detailed paper screening by independent researchers(MA and AM). For duplicate studies, we only considered the version with the most complete dataset and excluded the others.

### 2.3. Data Extraction and Quality Checks

Papers were retrieved from databases independently by the two researchers (MA and AM), and when there was any disagreement concerning eligibility criteria, it was resolved by the third author, RMG.

A data extraction sheet was developed based on extensive discussion between MA and AM to make sure all information obtained was significant enough for the review. Our primary outcome variable was meningitis, and the independent variable was diabetes as reported in previously published studies (see Table 1 for MESH terms). Items such as author names, year of population, and sample size (combined for control and treatment groups) were also extracted. We obtained the subjects' socioeconomic and physiological/clinical background data such as age and sex. It was decided priori that variables such as smoking, and alcoholism prevalence would be extracted. However, from our independent literature search, researchers were inconsistent with how they reported these variables (most did not report), and so we did not extract them. The country and region of the research were recorded. In situations where diabetes was not explicitly reported, a blood glucose level >200 mg/dL was considered a threshold for defining a diabetic patient. For studies that reported median, interquartile ranges, minimum, and maximum values instead of means for some covariates such as age, we adopted a series of formulas to transform them to means [33, 34]. For example, if the minimum, maximum, and median values were reported, the mean was approximated with the expression 1 as follows:

(1) $$\begin{array}{c} \text{Mean}=\;\frac{a+2m+b}{4}, \end{array}$$

where *a* is the minimum value, *m* is the median, and *b* is the maximum value. If the first quartile and the third quartiles were reported in addition to maximum and the minimum value, we estimated the mean with the expression 2 as follows:

(2) $$\begin{array}{c} \text{Mean}=\frac{a+\;2q_{1}+2m+\;2q_{3}+b}{8}, \end{array}$$

where *q*_1_ is the first quartile and *q*_3_ is the third quartile. If the median and the interquartile range was provided, we estimated the mean with the expression 3 as follows:

(3) $$\begin{array}{c} \text{Mean}=\;\frac{q_{1}+m+\;q_{3}}{3}. \end{array}$$

**Figure 1:**
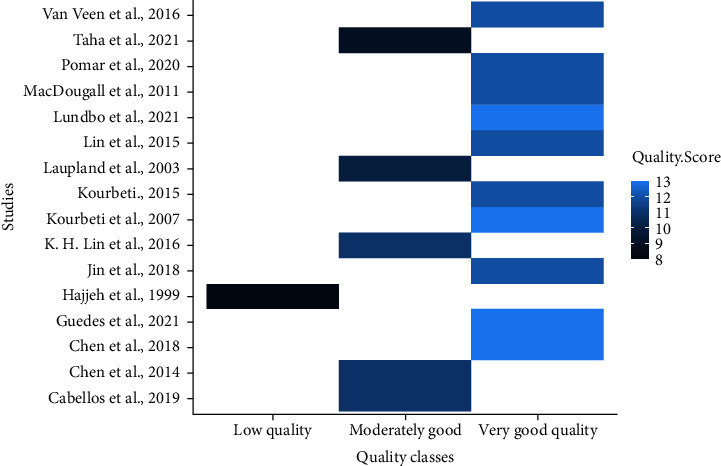
A plot indicating the quality score for individual studies.

To ensure inherent paper quality appraisal, a 12-point scoring system based on the Downs and Black checklist was adapted for the current study [35, 36]. Typically, these twelve items include the following: (1) if the research was clear about its objectives/aims/hypothesis, (2) if the research was clear about the study design it used, (3) if the study participants were representative, (4) whether the final recruits for the study were representative of the initial population (after some participants declined to participate), (5) if the study had a good sample size, (6) if the research stated incidence of missing data and how it was managed, (7) if the sociodemographic of participants were clearly stated, (8) if the association was quantified, it controlled for confounders (9), whether the research very clear about the results it reported, (10) if the study was explicit about how the variable of interest was measured (diabetes and meningitis), (11) the study reported any potential cases of biases or acknowledged limitations if it existed, and (12) if the outcome of interest for the research was explicitly stated. Other items known to be associated with quality such as inclusion and exclusion criteria for recruiting eligible study subjects, were assessed. We further considered if a study reported the etiology of the disease (meningitis). Moderate sample size in this study was defined as sample size ≥100 people. As result, we ended with a 16-point rating system instead of the 12-point system [35, 37]. Quality of the various papers based on the scoring system was divided into poor (0–7.99), good (8–11.99), and very good (12–16) (Figure 1).

### 2.4. Statistical Analysis

When the disease prevalence was reported separately based on sex or age, the average was computed and used for effect size estimation (assuming statistical independence). However, pooled odd ratios (OR) were calculated for different regions of the world (Europe, North America, and Asia) where data were enough using the OpenMEE software [38]. This is an open source software and suitable for advance meta-analysis, such as handling complex data structure and plotting high-resolution charts. Individual study ORs were estimated around 95% confidence intervals (CI) as well as the pooled OR. We assessed the total study (inherent study variance and between-study variance) variability using the *Q* and *I*^2^ statistic. *Q*-test of *p* value less than 0.05 was considered statistically significant variation across studies. Also, *I*^2^ greater than 50% was considered to have a meaningful heterogeneity in the pooled estimates of the association between diabetes and meningitis. Graphically, the funnel plot was used to detect publication bias, whereas the weighted Fail-Safe-N test [39] provided a quantitative metric. Acknowledging the limitation of the weighted Fail-Safe-N (FSN) test, we integratively assessed the publication bias using both the full plot and the FSN test. Before arriving at final models, we conducted residual/outlier tests to identify influential studies or outlier studies that may impact our pooled estimates using leave-one-out (LOO) analysis. To assess potential sources of significant heterogeneity across studies, we conducted moderator analysis using meta-regression/sub-group analysis. We performed meta-regression for covariates such as age of meningitis-infected participants, year of publication, sample size, study quality, and study design. Subgroup analysis was conducted for the flank of the world where the study was conducted, the study design and the major etiologic class of meningeal disease reported in the various studies. We relied on a mixed-effects model for the subgroup analysis where a random-effect model was applied to combine studies within each sub-group and a fixed-effect model was used to bring together subgroups and produce the estimate for the summary effect sizes (association between diabetes and meningitis). We excluded the prevalence of smoking and alcohol intake as potential moderators because most studies failed to report on them.

## 3. Results

### 3.1. Study Characteristics

**Figure 2:**
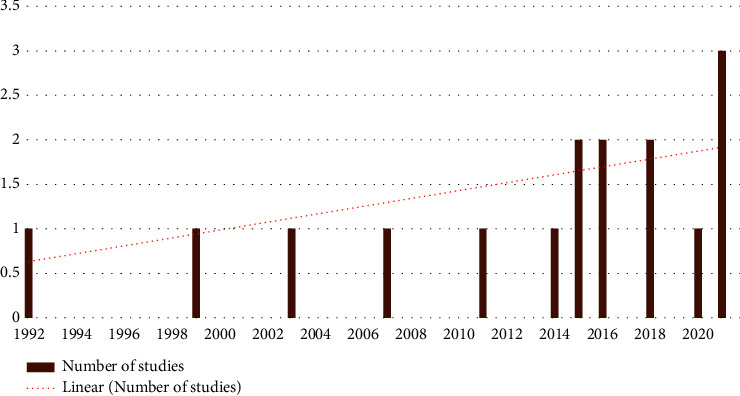
Studies conducted from 1997 to 2021.

There were no year restrictions on papers eligibility, even though almost all papers were between 2014 and 2021 (Figure 2), indicating that this health issue is only beginning to gain recognition among researchers.

**Figure 3:**
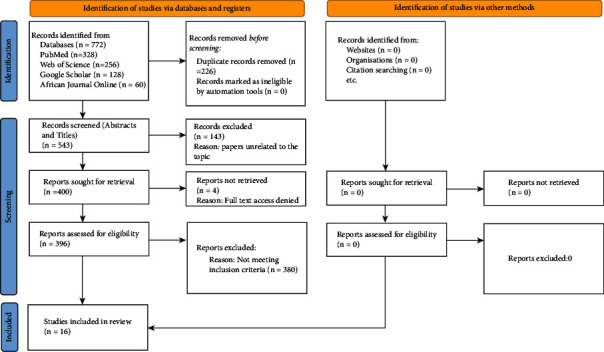
PRISMA 2020 flow diagram for new systematic reviews.

The individual study characteristics are presented in Table 2, and the flow charts for retrieving relevant papers for the meta-analysis are shown in Figure 3. The initial independent search yielded 772 articles and 229 of them were duplicates. Additional 147 papers were removed because their titles were not relevant to the subject of our research. We finally excluded an additional 380 papers which did not meet the inclusion criteria. The final analytic sample of 16 articles met our inclusion criteria.

**Figure 4:**
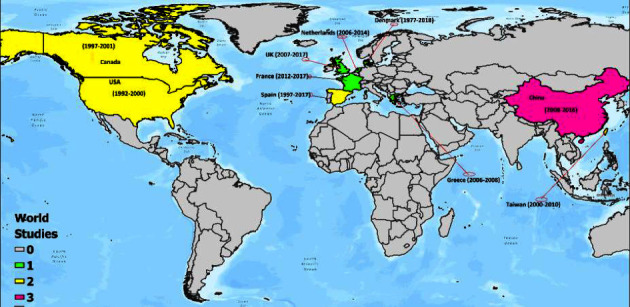
A map indicating the spatial distribution of papers conducted in different countries and the period of study.

The total number of meningeal disease cases was 16847 across all studies. Only one study was conducted before the year 2000 [46]. Four studies came from North America (USA = 2 and Canada = 2), 7 studies from Europe (UK = 1, Spain = 2, France = 1, Denmark = 1 and Netherlands = 1, and Greece = 1), and 5 articles from Asia (Taiwan = 2 and China = 3). No South American and African study reported the association between meningitis anddiabetes. Nonetheless, a significant number of research on meningitis or diabetes were identified in these global regions. The geographical distribution of studies conducted between 1999 and 2021 is indicated in Figure 4.

The longest follow-up period leading to the reportage on the association between diabetes and meningitis was conducted in Denmark (1977–2018) and Spain (1977–2017), and the shortest was in Greece (2006–2008) (Figure 4). The average age of people who suffered from meningitis infection was 47.76 years (95% CI: 40.93, 54.59). About 53% (*n* = 9) of the papers used retrospective study designs and approximately 35% (*n* = 6) used prospective study designs. Two studies used population-based active surveillance cohort [47] and observational cohort [4] study designs. Two major etiologic classes were fungus and bacteria, even though bacterial meningitis was the dominant: Bacterial meningitis (*n* = 10 studies; 59%) and fungal meningitis (*n* = 7 studies; 41%) (Table 2).

**Figure 5:**
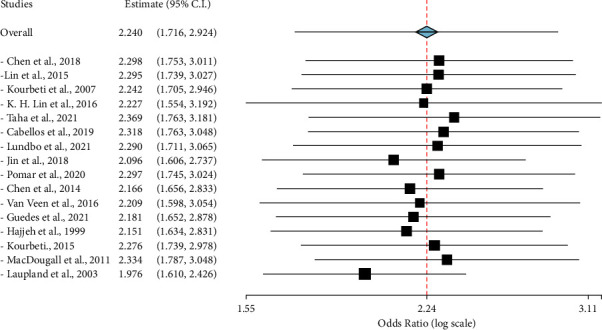
The summary evidence of the impact of diabetes on developing meningitis. The data is illustrated for each study effect size represented by the black box, and the overall summary, with their 95% confidence intervals.

Diabetes has been identified as a potential risk factor for meningitis. However, studies reporting on such evidence are significantly limited. The overall pooled OR and point estimate for the effective sizes for various studie are shown in Figure 5. The ORs were not statistically significant in [5, 26, 29, 38, 46] (Figure 5). Based on the random effective model (using the Der-Simonian and Laird model), the overall effect size (ES) showing the diabetes-meningitis link was OR = 2.240 (95% CI: 1.716, 2.924; *p* < 0.001) indicating that diabetes increases the risk of developing meningitis by two-fold. However, there was significant heterogeneity of ES among individual studies (*I*^2^ = 86.95%; *p* < 0.001). The possible sources of heterogeneity were, therefore, investigated.

**Figure 6:**
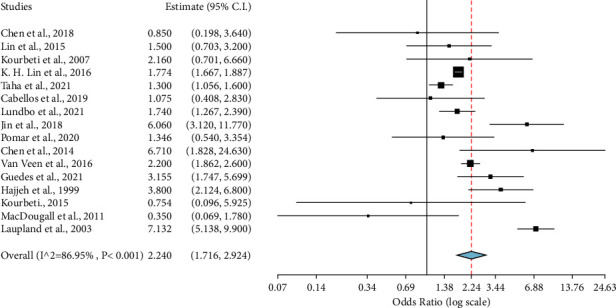
A leave-one-out examination indicating how each study influences the overall effect size and suggesting the association between diabetes and the risk of meningitis. The figure is illustrated to present the new effect size, provided each study was removed. The horizontal lines show the confidence intervals at a 95% significance level.

**Figure 7:**
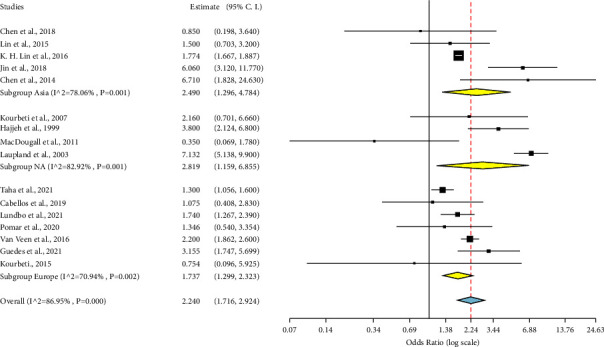
The synthesized evidence of the impact of diabetes on meningitis risk for various continents. NA means North America. The data is illustrated for each study effect size, represented by the black box and the overall summary; with their 95% confidence intervals.

We did leave-one-out (LOO) analysis to assess the overall influence of individual studies on the general ES, and the significance of the risk remained apparent (Figure 6), suggesting that the pooled ES was relatively stable and was not determined by only one individual study. Our subgroup analysis for continental effect estimates is shown in (Figure 7). With regional subgroup analysis of Europe OR = 1.737 (95% CI: 1.299 2.323; *p* < 0.001), Asia OR = 2.490 (95% CI: 1.296, 4.784; *p* = 0.006), and North America OR = 2.819 (95% CI: 1.159, 6.855, *p* = 0.022), diabetes increased the risk of developing meningitis by nearly two-fold. However, there was significant heterogeneity among studies from all these continents: Europe (*I*^2^ = 70.94%; *p* = 0.002), Asia (*I*^2^ = 78.06% *p* = 0.002), and North America (*I*^2^ = 82.92%; *p* < 0.001). Both study designs; retrospective OR of 2.044 (1.335, 3.131; *p* < 0.001: *I*^2^ = 76.18; *p* = 0.001) and prospective study designs OR of 2.428 (95% CI: 1.599, 3.689; *p* < 0.001: *I*^2^ = 92.34; *p* < 0.001) found statistically significant association between diabetes and meningitis. This indicates that even if we divide studies between these separate study designs, diabetes will remain a significant risk factor.

**Figure 8:**
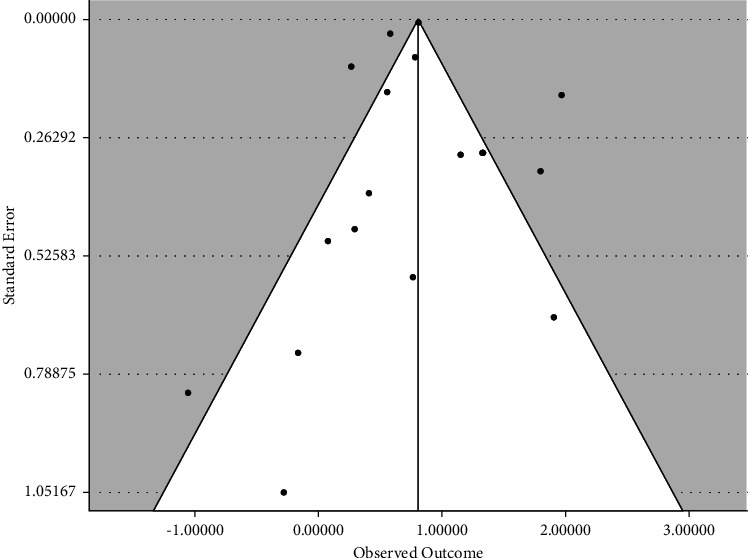
Funnel plot showing the symmetric nature study distribution with respect to their outcome and standard errors.

We also found that diabetes increased the risk of meningitis regardless of the etiological class: fungal meningitis OR = 2.290 (95% CI: 1.033, 5.075; *p*=0.041) and bacterial meningitis OR = 2.190 (95% CI: 1.567, 3.060; *p* < 0.001) even though the strength of association seems to be marked among cases caused by bacteria. Our publication bias diagnostics were conducted by using the funnel plot (Figure 8). Our weighted fail-safe*N* test (FNS = 1023; target ES = 0.01(logscale) suggested that additional 1023 studies may be needed to bring down the pooled ES of 2.240 to OR ≤1. Our funnel plot confirmed this diagnostic, as it showed moderate symmetry among studies. A univariate meta-regression (using maximum likelihood for the coefficient estimation) based on covariates indicated a significant variation in the covariate effects on the ES. Particularly, age explained 10.96% of the overall heterogeneity found, even though the covariate effect (*Q* = 0.181; *p*=0.542) was not statistically significant. Nevertheless, the positive regression coefficient (*β* = 0.004; *p*=0.542) may suggest that increasing age will determine the risk of meningitis. The study sample size explained 7.83% of the heterogeneity, although the covariate effect was also not statistically significant (*Q* = 1.495; *p*=0.221). However, the subgroup analysis among studies with >500 or <500 sample size, the ES was significant in larger sample sizes. For example, association was significant for studies with sample size >500: OR = 2.283 (95% CI: 1.71, 3.05; *p* < 0.001) but insignificant for sample size <500: OR = 1.692 (95% CI: 0.65, 4.39; *p*=0.280). Study quality score was marginally significant (*r*^2^ = 26.08%: *Q* = 2.891; *p*=0.089). Study design explained approximately 0% of the heterogeneity (*r*^2^ = 0%: *Q* = 0.047; *p*=0.828). The year of publication was the most significant covariate that explained a significant portion of the heterogeneity in the effect sizes (*r*^2^ = 40.42%), and its covariate effect was statistically significant (*Q* = 11.31; *p* < 0.001) Table 3.

Our multivariate meta-regression analysis of potential predictors of heterogeneity revealed that age, sample size, year of publication, and assigned quality score together explained 79.49% of the total heterogeneity among studies, and their covariate effect was statistically significant (*R*^2^ = 0.79.49; *Q* = 25.35; *p* < 0.001) (Table 3).

## 4. Discussion

Infectious diseases remain a significant threat to the wellbeing of people and the economies of countries worldwide [7, 50–55]. Our study included 16847 meningitis cases with 7 prospective and 9 retrospective study designs. Meningitis infection is a global threat to the wellbeing of people. If left untreated, the meningitis fatality rate can exceed 70% [50]. Generally, meningitis mortality has declined by 21% between 1990 and 2016, yet about 690,000 people died in 2015 alone [12], which makes it a significant public health challenge. Even among survivors of meningitis, one in five may develop permanent sequelae, including cranial nerve palsies, hydrocephalus, seizures, hemiparesis, and visual and hearing impairment [1, 7].

Accumulating research provides evidence on the pathophysiology of the disease, but little knowledge exists on how its prevalence and mortality are further amplified by other coinfections such as diabetes. However, diabetes has been noted as an independent risk factor for multiple infections, including tuberculosis and pneumonia [20, 21] and a substantial factor in hospitalization [20]. Nevertheless, the role of diabetes in the development of meningitis is less acknowledged. This may account for the smaller number of studies discovered during our meta-analysis. Our review discovered that diabetes increases the risk of developing meningitis by two-fold. In addition, there was a significant degree of heterogeneity among individual studies. The risk level found corroborates with a population-based prospective study [27], suggesting that people with diabetes were twice more likely to have bacterial meningitis than those without diabetes. Such relationships in our current review remain fairly constant when compared across continents. In Taiwan, for instance, the incidence of diabetes in people with bacterial meningitis can be as high as 39% [56]. Apart from Asia, Europe, and North America, we found no studies from any other part of the world, including Africa. To our knowledge, this is the first meta-analysis synthesizing the association between diabetes and meningitis. Empirical evidence is limited, with most evolving papers being short case reports. More studies are needed from a considerable number of countries and from diverse socioeconomic and physiological backgrounds. Lack of such studies in Africa may deepen the burden of meningitis and diabetes [57–59]. It is estimated that the prevalence of type-2 diabetes will increase by 134% between 2019 and 2045 in Africa [60–62]. Concurrently, 29 countries in Africa labelled “meningitis belt” are at persistent risk of meningitis [63]. The co-existence of these two diseases may impose a further burden on the scarce economic and health resources as well as the quality of life of the population at risk. There was an evidence of insignificant publication bias in our study, as indicated by the funnel plot.

However, the complex relationship between diabetes and meningitis was not broadly explored in variousstudies. Such an intricate mechanism remains largely unclear. Nevertheless, few studies have investigated this connection. Hyperglycemia, which is strongly associated with diabetes [64], is related to negative outcomes in multiple infections, including neurological disorders such as stroke, sepsis, and head injury [65]. Furthermore, diabetes, characterized by insulin resistance may result in a serious immunocompromised state of people [22]. As a result, there is a greater propensity for decreased cell-mediated immune activities and dysfunctioning of the polymorphonuclear leukocytes, monocytes, and lymphocytes. These conditions have been associated with the magnitude and persistency of hyperglycaemia in patients [26, 27, 66]. This extreme immunosuppression may explain the linkage between diabetes and meningitis. Additionally, the cumulative concentration of sugar in the blood due to insulin resistance or less production of insulin may provide a conductive environment for fungal or bacterial growth in the body, thus giving more room for the development of bacterial infections such as bacterial meningitis. This may relate to the widespread incidence of bacterial meningitis among people with diabetes and severe hyperglycaemia [31, 56, 65]. Nonetheless, the association is not consistent across studies. In our meta-analysis for instance [5, 26, 29, 38, 46], we found no statistically significant association between diabetes and meningitis even though the pull effect size indicated an increased risk.

It should, however, be noted that most of the studies that found no association between diabetes and meningitis were conducted in hospital settings, where other factors such as intraoperative cerebrospinal fluid leakage may expose patients to meningitis. While our meta-regression suggested that age, year of publication, study quality, and study design explained 79% of the finding's heterogeneity across studies, other factors such as smoking, alcoholism, malnutrition, and comorbidities still remain significant [6]. In effect, the inconsistent association between meningitis and diabetes may be further explained by: (1) underlying comorbidities, (2) personal lifestyle choices and nutrition, (3) aetiologic complexity of meningitis itself, and (4) the environment where the infection is acquired. For example, the regional variation in the etiology of bacterial meningitis alone is significantly complex, and since the evolution of multiple vaccines, its etiologic spectrum has changed considerably [31]. Considering ecology and meningitis, most of the hospital-based studies considered in our meta-analysis found no statistical evidence for a positive association. These cases were hospital-based where patients had undergone surgery or medical treatment. Therefore, other factors such as multiple operations, pre-existing comorbidities, intraoperative cerebrospinal fluid leaks, and endoscopic approach used in the surgery may be stronger correlates of meningitis [44, 67]. Factors such as active/passive smoking and alcohol abuse have also been noted as independent risk factors for meningitis [15, 16, 19].

Although our current meta-analysis found no differences in the effect sizes between fungal and bacterial meningitis, the broader grouping into bacteria and fungus may sometimes mask the finer differences in how specific etiologic agents of these broader groups moderate the risk. For example, cryptococcal meningitis which is fungal has been discovered to be most prevalent among people living with HIV (PLHIV) whereas, among the general population, bacterial meningitis presents the greatest meningeal disease burden, currently infecting 1.2 million annually around the world. This may explain why about 60% of studies were based on bacterial meningitis. In one study, it was noted that diabetes mellitus increased the risk of *S. pneumoniae,* followed by *L. monocytogenes*. Conversely, age, pregnancy, an underlying cancer condition, and immunosuppression were significant predictors of *L. monocytogenes* instead of *S. pneumoniae* [31]. The etiological complexity must therefore be accorded the needed attention when measuring the association between diabetes and meningitis. Also, while our current study suggests an increased risk of meningitis due to diabetes independent of study design and etiological class, we particularly recommend more primary studies from a multiplicity of situations/settings. These may include (1) how diabetes (type 1 and 2) predicts the risk among several meningitis subtypes and (2) how such an association is moderated by lifestyle choices, nutrition, socioeconomic conditions, and comorbidities.

Our study has a major strength by increasing the statistical power due to the larger sample size as compared to individual studies. For example, subgroup analysis between studies based on sample size indicated that research with a sample size greater than 500 found a statistically significant association (*p* < 0.001) while research with a sample size less than 500 found no association (*p*=0.280). This suggests that our conclusion is based on larger sample size studies with higher statistical power, which implies how significant our meta-analysis is in increasing the statistical strength of the association. However, there are some limitations. Firstly, some studies evaluated the association based on retrospective case-control study designs which may be subjected to selection and recall biases [64]. Therefore, larger cohort studies are needed to confirm our conclusions. Secondly, some studies relied on standardized questionnaires and International Statistical Classification of Diseases (ICD) codes to identify meningitis and diabetes which may also have some elements of misclassification and errors. Nevertheless, most research followed medically approved means of defining and confirming meningitis and diabetes; we, therefore, do not expect significant bias in our pooled estimate. It is also worth mentioning that some studies (including our meta-analysis) considered diabetes in general. But it is proven that types 1 and 2 diabetes may influence certain diseases differently since they are pathophysiologically different [68]. For example, type 1 diabetes is commonly associated with reactive autoimmune activities, whereas type-2 diabetes which is highly related to obesity, affects tissues through chronic, systemic, low-grade inflammation rather than reactive autoimmune [69]. Studies providing a distinctive analysis of how these two types affect meningitis disease in general will be useful.

## 5. Conclusions

We conducted a meta-analysis involving 16847 cases to investigate the relationship between diabetes and the risk of developing meningitis. Our study indicated that diabetes increased meningitis risks by more than two-fold. This risk is very significant across all global regions included in the study (North America, Europe, and Asia). While this finding corroborates with some other studies, no research was found from South America and Africa, which obviously are the most plagued in terms of meningitis and diabetes disease burden. The findings suggest that attention should be drawn to this research theme in Africa and other LMICs. Crucially, studies focusing on detailed subtypes of meningitis and their association with different classes of diabetes will be helpful to confirm our findings and provide comprehensive evidence for the pathology and etiology of meningitis.

## Figures and Tables

**Table 1:** Medical subject headings (MESH) terms used for extracting papers.

The subject	The MESH terms
Meningitis	“Arachnoiditis meningitis,” “bacterial meningitis,” “*E. coli* meningitis,” “listeria meningitis,” “meningococcal meningitis,” “pneumococcal meningitis,” “cryptococcal” “lymphocytic choriomeningitis,” “meningoencephalitis”
Diabetes	“Diabetes mellitus,” “type 2 diabetes mellitus,” “lipoatrophic diabetes,” “gestational donohue syndrome,” “latent autoimmune diabetes.”

**Table 2:** Study characteristics and the quality score assigned. All columns' values under disease etiology indicated as “Multiple” represent studies that reported finding both negative and negative grams but did not specify these pathogens. Same for sections indicated “General”.

Study	Country	Study period	Analytic method	Disease etiology	Study design	Quality score	Major etiology	Definition of meningitis
[40]	China	2014-2015	Multivariate and univariate logistic	Multiple	Retrospective	13	General	Meningitis was defined by: (1) organisms cultured from CSF; (2) at least one of these signs without identified cause: Fever (>38°C), headache, stiff neck, meningeal signs, irritability, and if analysis was conducted antemortem, attending physician instituted appropriate antimicrobial therapy, and at least one of the following: (a) Elevated white blood cell count, a rise in protein level in the CSF, and/or attenuated glucose level in CSF, (b) positive antigen test of CSF or blood; and (c) investigative single antibody titer (IgM) or an increase in paired sera (IgG) for pathogen by four-fold

[28]	Taiwan	2002–2010	Conditional logistic regression	*C. neoformans meningitis, C. neoformans fungemia*	Retrospective cas-control	12	Fungal	Cerebrospinal fluid [CSF]or blood culture positive culture n

[29]	USA	1996–2000	Logistic regression	*Coagulase-negative staphylococci, Acinetobacter calcoaceticus, Pseudomonas aeruginosa, Serratia marcescens, Serratia species, Haemophilus influenzae*	Retrospective	13	Bacteria	Meningitis was defined through gram stain, CSF culture or both, CSF leukocytosis with elevated protein concentration and reduced glucose level or both. Fever or nuchal rigidity with unknown cause or patients under antibiotic treatment prescribed by a physician.

[41]	Taiwan	2000–2010	Logistic regression	Cryptococcal meningitis	Prospective case-control	11	Fungal	Definition of meningitis: Cryptococcosis (ICD-9 117.5) or cryptococcal meningitis (ICD-9 321.0)

[42]	France	2012–2017	Logistic regression	Invasive meningococcal disease	Case-control	9	Bacteria	Meningitis was defined according to ICD-10 diagnostic code (A39.0 to A39.9) criteria

[4]	Spain	1977–2013	Simple linear regressions	*Neisseria meningitidis, Streptococcus pneumoniae, Listeria monocytogenes*	Observational cohort study	11	Bacteria	Meningitis was defined through the following means: a Positive CSF culture, the occurrence of negative cultures when Gram negative diplococci was found from the CSF stain or when patients showed incidence of severe bacterial meningitis which is medically confirmed

[43]	Denmark	1977–2018	Logistic regression	Meningococcal serogroups B&C	Case-control	13	Bacteria	Positive CSF culture, positive antigen tests, on Gram's stain of CSF

[44]	Taiwan	2012–2016	Logistic regression	Coagulase-negative*Staphylococcus, Staphylococcus aureus, Streptococcus pneumoniae, Viridans* group*, streptococci, Enterococcus faecalis, Corynebacterium, Micrococcus luteus, Gemella morbillorum, Klebsiella pneumoniae, Enterobacter aerogenes, Pseudomonas aeruginosa, Acinetobacter baumannii, Escherichia coli, Bacteroides fragilis, Citrobacter freundii, Morganella morganii, Enterobacter cloacae, Cryptococcus neoformans*	Retrospective observational	12	Bacteria	Positive organism CSF culture, and at least one of the following signs or symptoms where no other recognized cause was observed: Fever (>38°^C^), headache, stiff neck, meningeal signs, cranial nerve signs, or irritability

[31]	Spain	1982–2017	Logistic regression	*Neisseria meningitidis, Streptococcus pneumoniae, Listeria monocytogenes, Gram-negative bacilli*	Prospective observational cohort study	12	Bacteria	Meningitis was defined as a diagnostic outcome of positive CSF culture and positive antigen tests. Any negative culture was further confirmed through CSF neutrophilic pleocytosis (=>100 neutrophils/cu mm or decreased CSF glucose (defined as CSF/blood glucose ratio <0.40) or elevated CSF proteins >0.5 g/l (for unknown etiology)

[30]	China	Jan-December 2008	Logistic regression	*Acinetobacter baumannii, Enterococcus* sp*, Streptococcus intermedius and Klebsiella pneumonia*	Retrospective cohort study	11	Bacteria	Patients had meningitis if they had one of these unknown indications: (Fever >38°c), meningeal signs, elevated white cell count, increased protein, or reduced glucose in the CSF. Organism identified on Gram's stain of CSF, antigen test, positive blood culture, a prognosis of one antibody titer (IgM) or an elevation in paired sera (IgG) for pathogen by four-fold

[27]	Netherlands	2006–2014	Logistic regression	*Streptococcus pneumoniae, Neisseria meningitidis, Listeria monocytogenes*	Prospective cohort	12	Bacteria	Bacterial meningitis was defined as having a positive cerebrospinal fluid culture, or a mix of positive blood culture without a significant pathogen, or a positive PCR result for streptococcus *pneumoniae* or neisseria meningitis with at least one cerebrospinal fluid finding predictive bacterial meningitis of a CSF of leukocyte counts >2000 cells/mm^3^, polymorphonuclear leukocyte count >1180 cells/mm3, glucose level <1.9 mmol/L, protein level >2 g/L, or CSF/blood glucose ratio <0.23

[45]	UK	2007–2017	Multivariate cox models	*Neisseria meningitidis*	Retrospective observational cohort study	13	Bacteria	Not reported

[46]	USA	1992–1994	Conditional logistic regression	*Cryptococcus neoformans*	Prospective study	8	Fungal	Positive culture for C. neoformans for any body part; detection of cryptococcal antigen in the blood, cerebrospinal fluid, or urine; or histopathologic findings consistent with cryptococcosis

[47]	Canada	1999-2000	Logistic regression	*Staphylococcus aureus, Enterococcus, Streptococcus, Clostridium butyricum, Candida albicans*	A population-basedactive-surveillance cohort design	10	Fungal	CSF and blood culture, pleural or synovial fluid, or aseptically obtained deep-tissue aspirates or surgical-tissue samples

[48]	Greece	2006–2008	Multivariate logistic regression, mantel-haenszel test	*Acinetobacter* spp., *Klebsiella* spp., *Pseudomonas aeruginosa, Enterobacter cloaceae, Proteus mirabilis*	Prospective study design	12	Bacteria	CSF culture, signs of: fever, headache, stiff neck, meningeal and cranial nerves signs or irritability (if diagnosis was made antemortem) antimicrobial therapy, increased WBC counts, increased protein level or increased level of glucose in the CSF, organisms seen on Gram stain of CSF; organisms cultured from blood; positive antigen test of CSF, blood, or urine; diagnostic single antibody titer (IgM) or 4-fold increase in paired sera (IgG) for pathogen

[49]	Canada	1997–2001	Logistic regression	*Cryptococcus gattii, Cryptococcus neoformans*	Case-control study	12	Fungal	CSF and/or blood culture and classification of diseases, 9^th^ revision (ICD-9), code 117.5 (cryptococcosis)

**Table 3:** Various variables and their coefficients generated through multivariate meta-regression.

Variable	Beta	Lower conf	Upper conf	*p* value
Age	−0.007	−0.017	−0.04	0.228
Sample size	0.000	0.000	0.000	0.441
Quality score	0.133	0.043	0.224	0.004^*∗∗*^
Publication year	−0.053	−0.079	−0.028	<0.001^*∗∗∗*^

## Data Availability

The data supporting this Systematic Review/Meta-Analysis are from previously reported studies and datasets, which have been cited. The processed data are available upon request from the corresponding author.
